# Supplementary figures and images for: A human forebrain organoid model reveals the essential function of GTF2IRD1-TTR-ERK axis for the neurodevelopmental deficits of Williams syndrome
Source: eLife. 2024 Dec 13;13:RP98081. doi: 10.7554/eLife.98081 (PMC11643624; doi:10.7554/eLife.98081)

Figure 3J

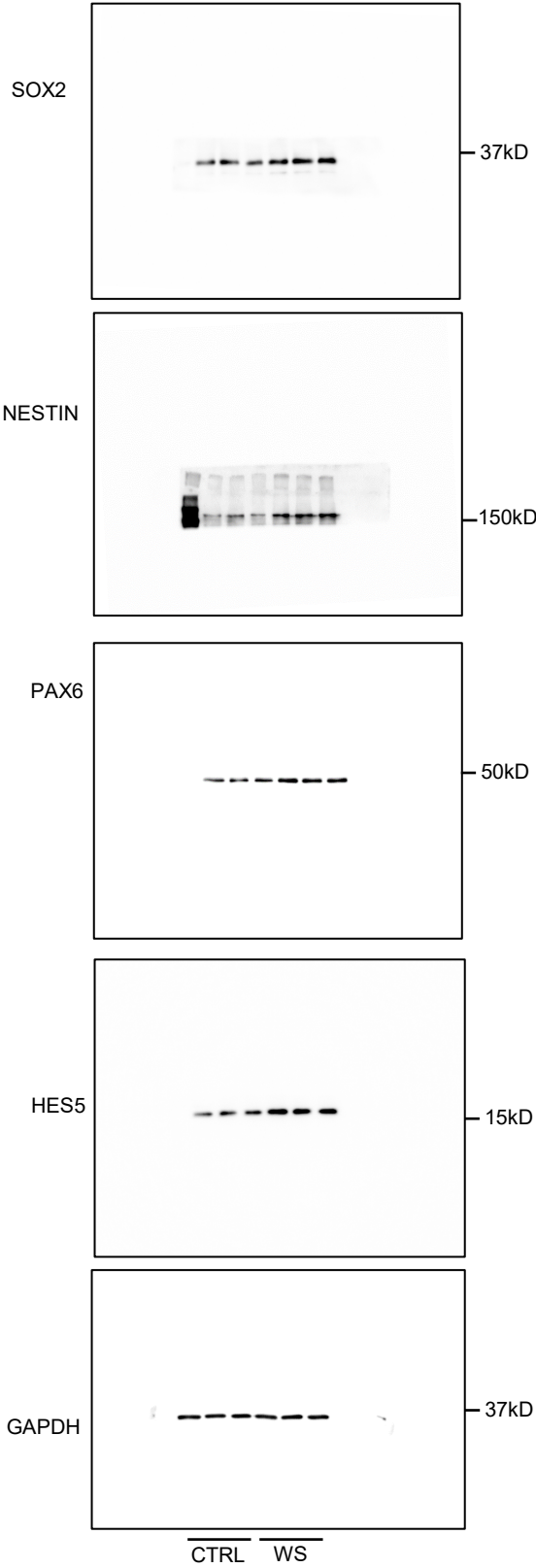

Figure 3O

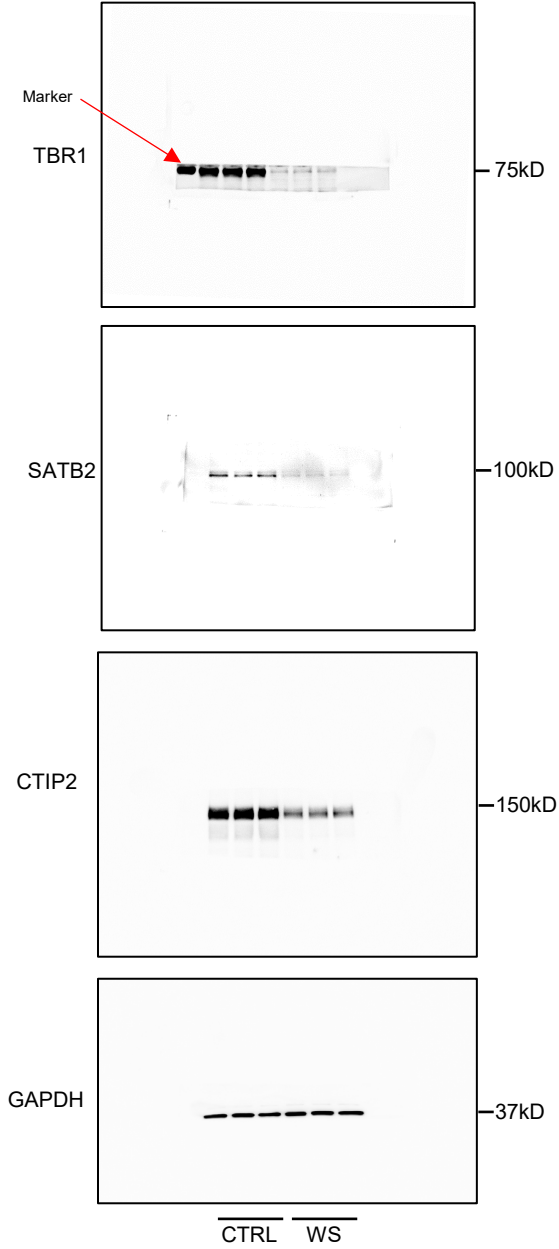

Supplement: Figure 3—source data 1. [file elife-98081-fig3-data1.zip › Figure 3-source data 1.pdf]

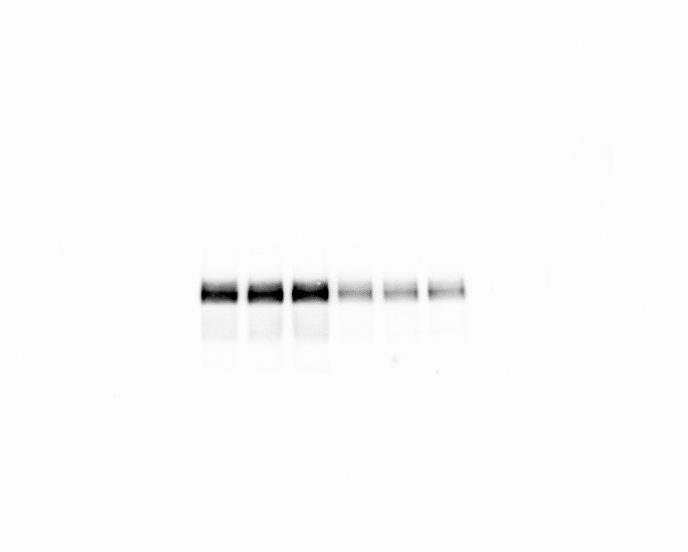

Supplement: Figure 3—source data 2. [file elife-98081-fig3-data2.zip › Fig3-source data 2/O/CTIP2.tif]

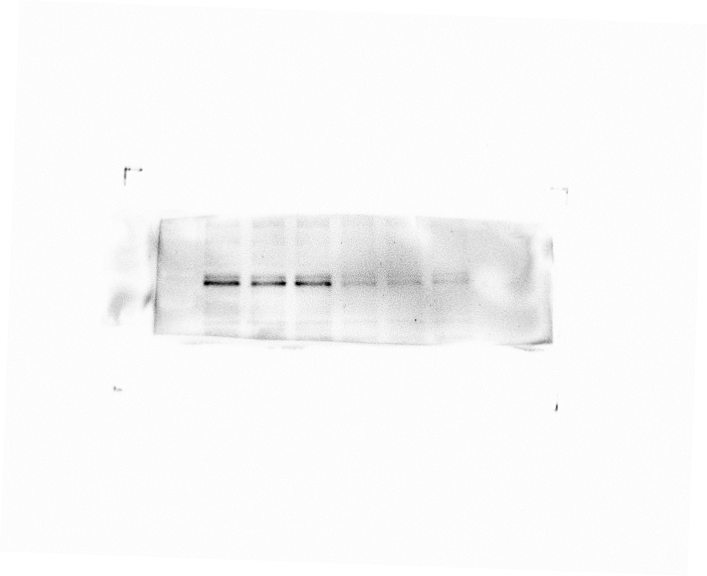

Supplement: Figure 3—source data 2. [file elife-98081-fig3-data2.zip › Fig3-source data 2/O/SATB2.tif]

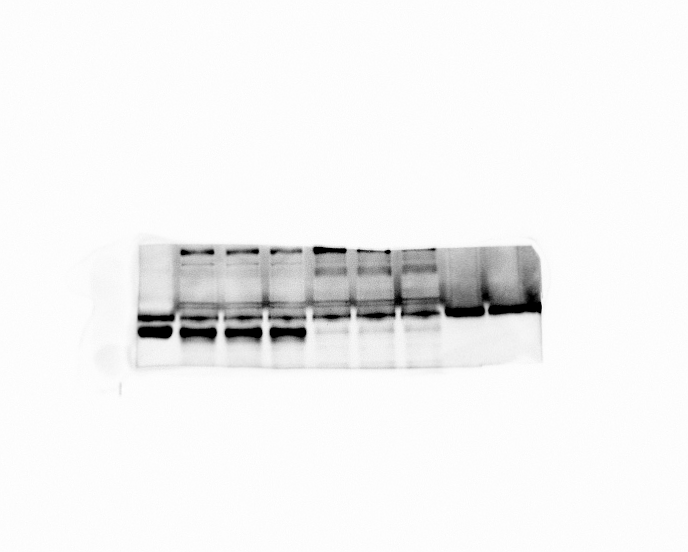

Supplement: Figure 3—source data 2. [file elife-98081-fig3-data2.zip › Fig3-source data 2/O/TBR1.tif]

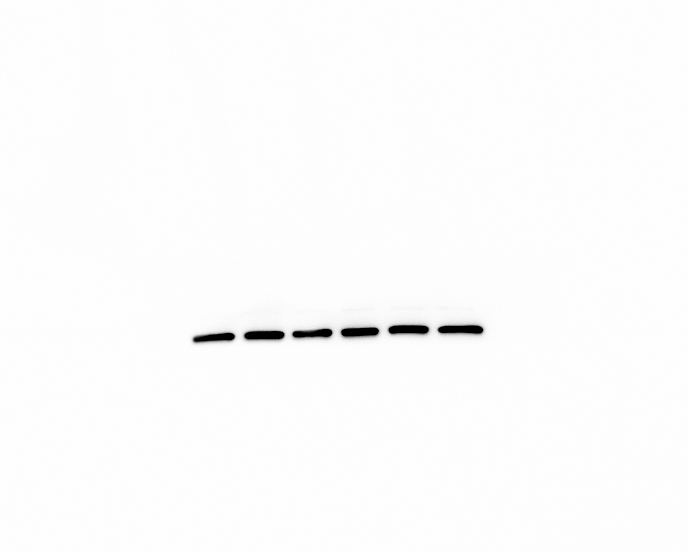

Supplement: Figure 3—source data 2. [file elife-98081-fig3-data2.zip › Fig3-source data 2/O/GAPDH.tif]

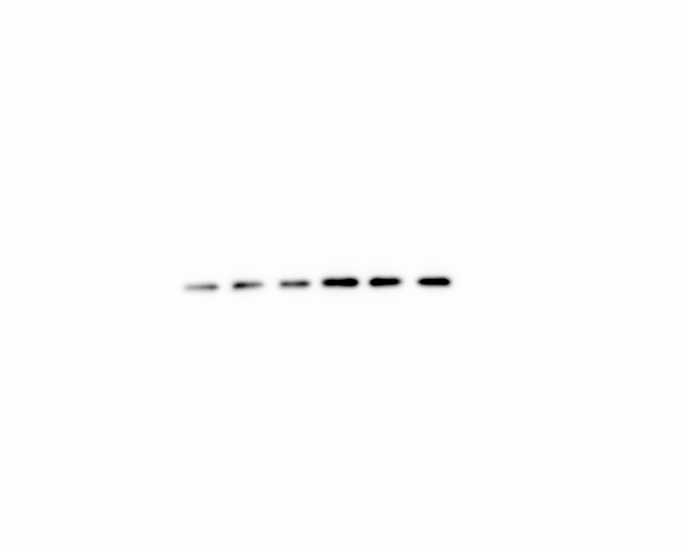

Supplement: Figure 3—source data 2. [file elife-98081-fig3-data2.zip › Fig3-source data 2/J/HES5.tif]

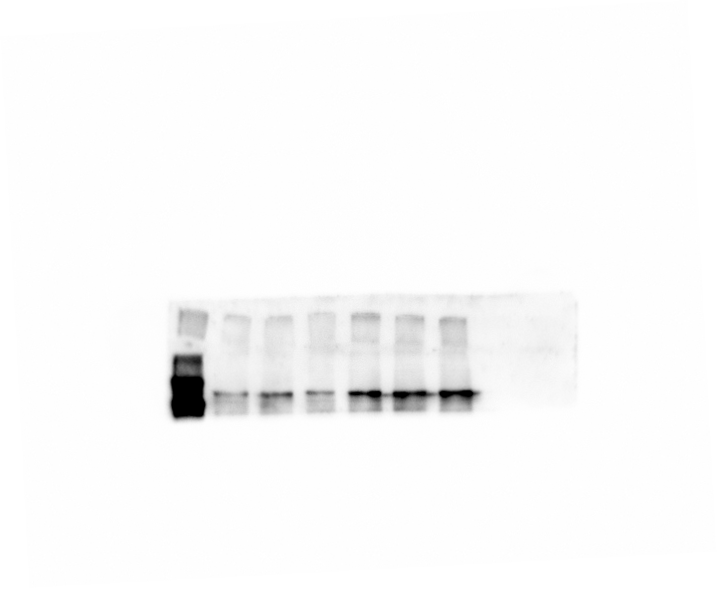

Supplement: Figure 3—source data 2. [file elife-98081-fig3-data2.zip › Fig3-source data 2/J/NESTIN.tif]

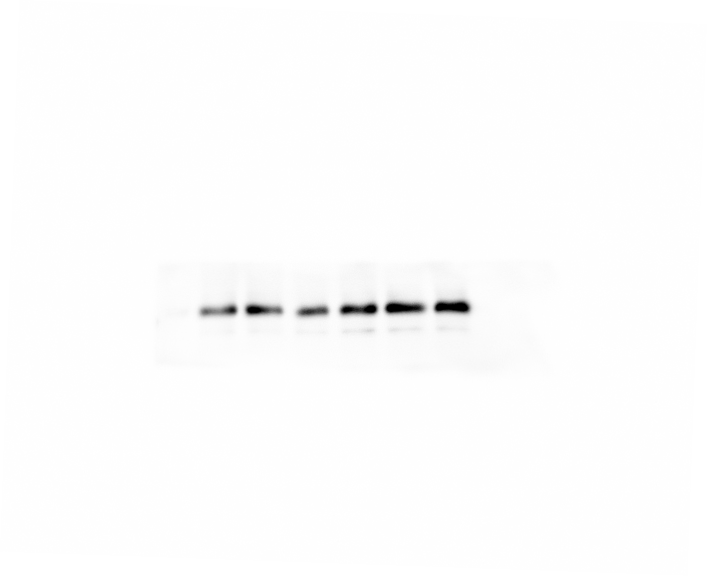

Supplement: Figure 3—source data 2. [file elife-98081-fig3-data2.zip › Fig3-source data 2/J/SOX2.tif]

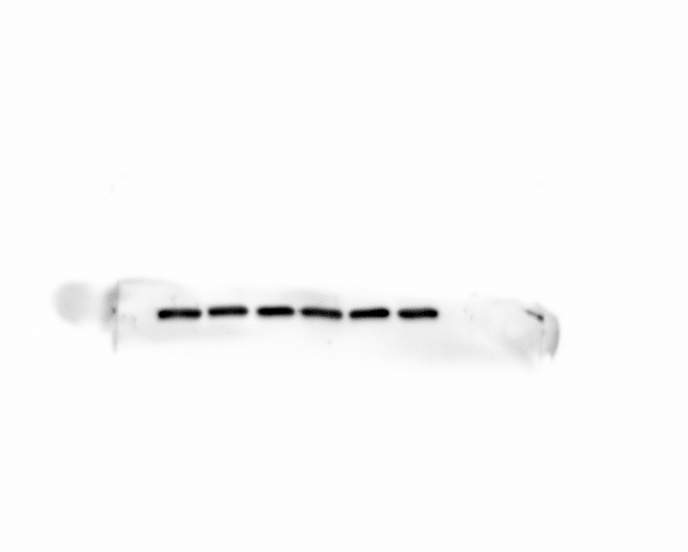

Supplement: Figure 3—source data 2. [file elife-98081-fig3-data2.zip › Fig3-source data 2/J/GAPDH.tif]

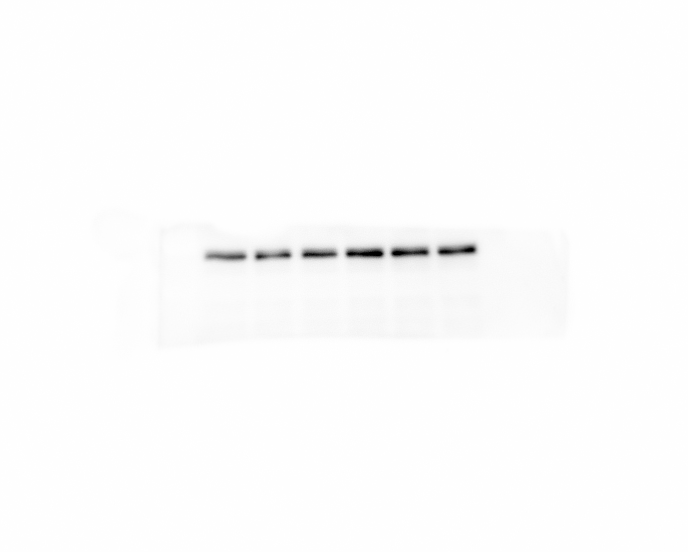

Supplement: Figure 3—source data 2. [file elife-98081-fig3-data2.zip › Fig3-source data 2/J/PAX6.tif]

Figure 6F

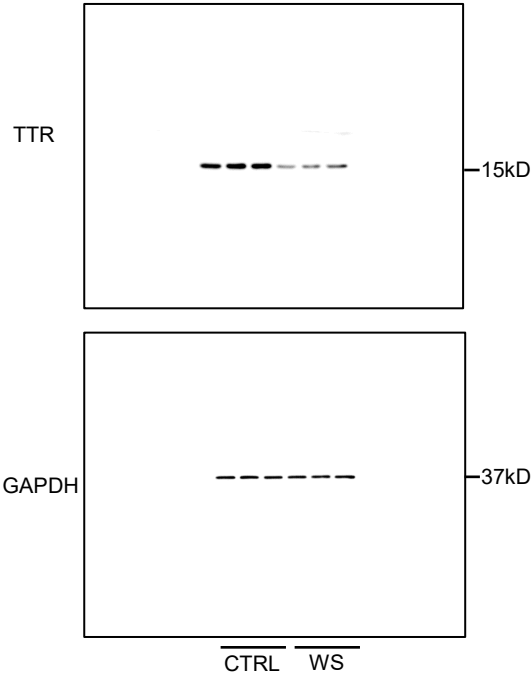

Figure 6I

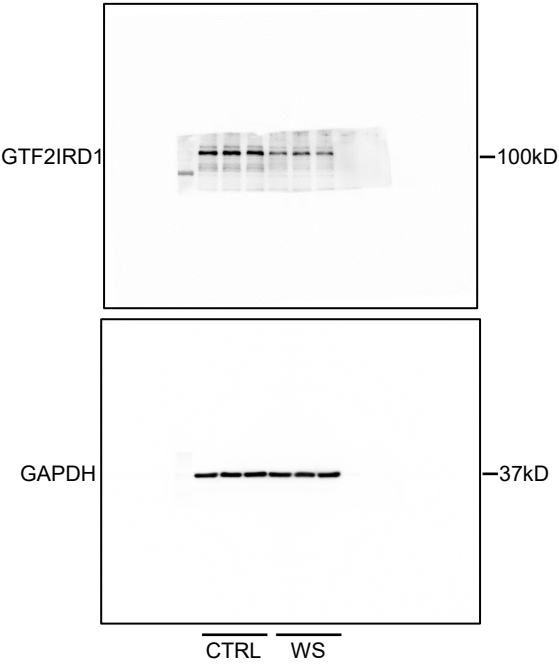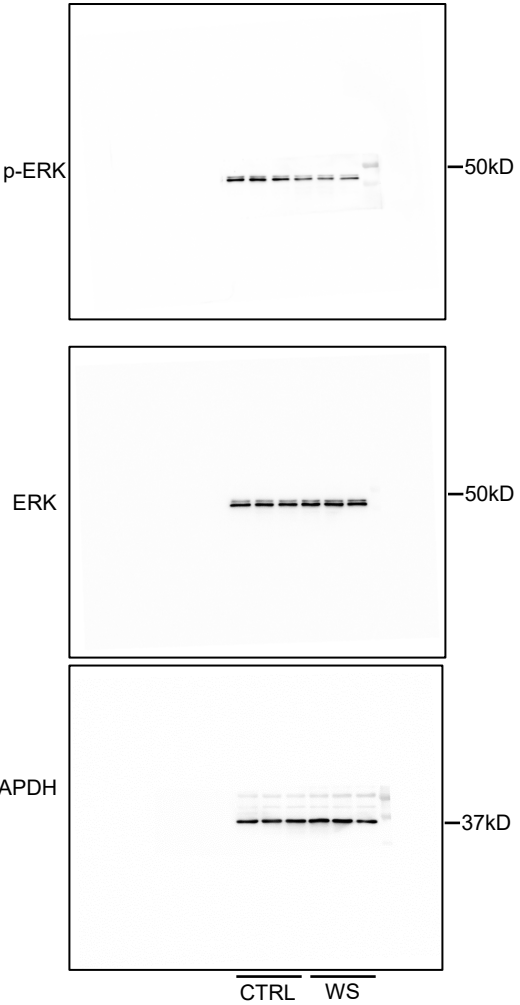

Supplement: Figure 6—source data 1. [file elife-98081-fig6-data1.zip › Figure 6-source data 1.pdf]

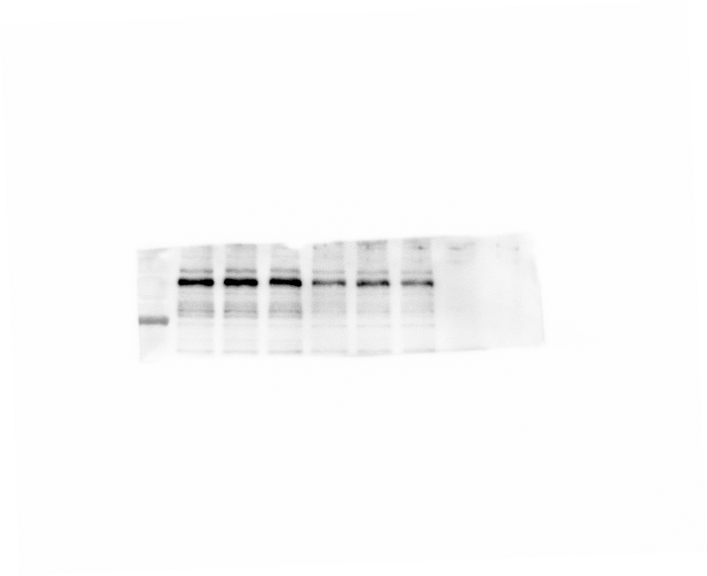

Supplement: Figure 6—source data 2. [file elife-98081-fig6-data2.zip › Figure6-source data 2/I/GTF2IRD1.tif]

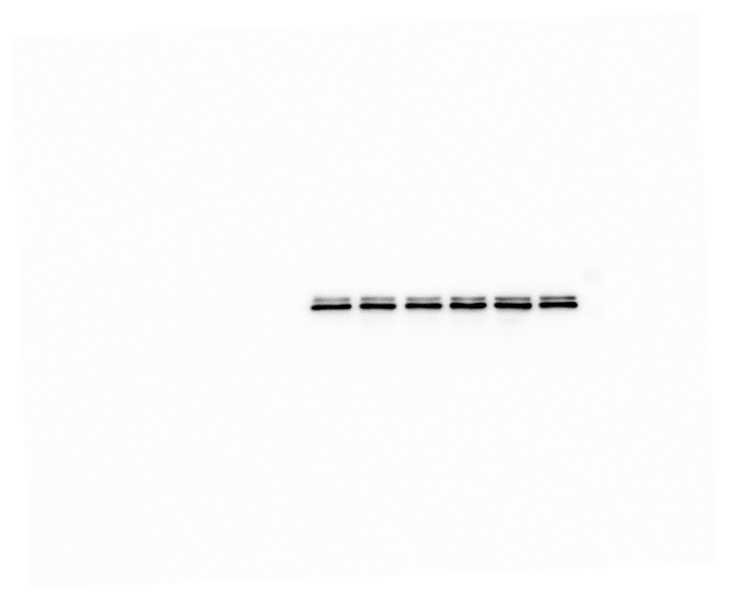

Supplement: Figure 6—source data 2. [file elife-98081-fig6-data2.zip › Figure6-source data 2/I/ERK.tif]

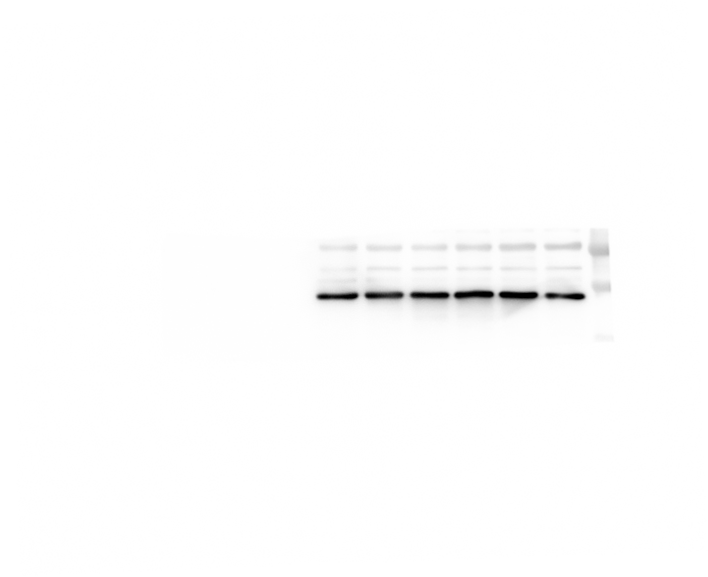

Supplement: Figure 6—source data 2. [file elife-98081-fig6-data2.zip › Figure6-source data 2/I/GAPDH-ERK.tif]

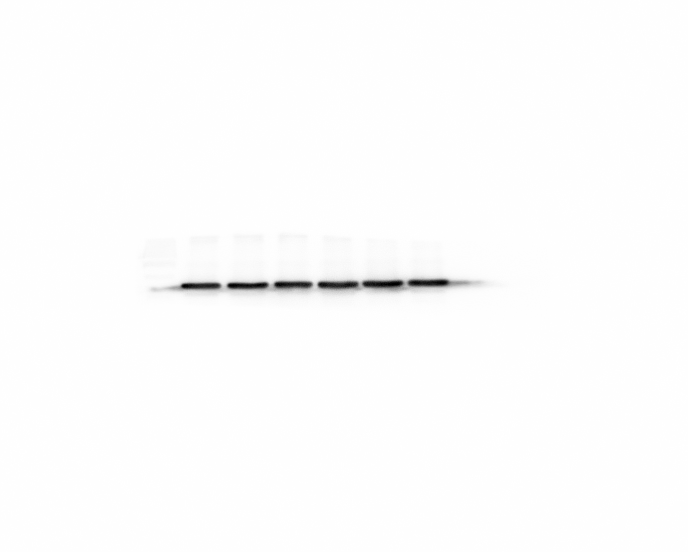

Supplement: Figure 6—source data 2. [file elife-98081-fig6-data2.zip › Figure6-source data 2/I/GAPDH-GTF2IRD1.tif]

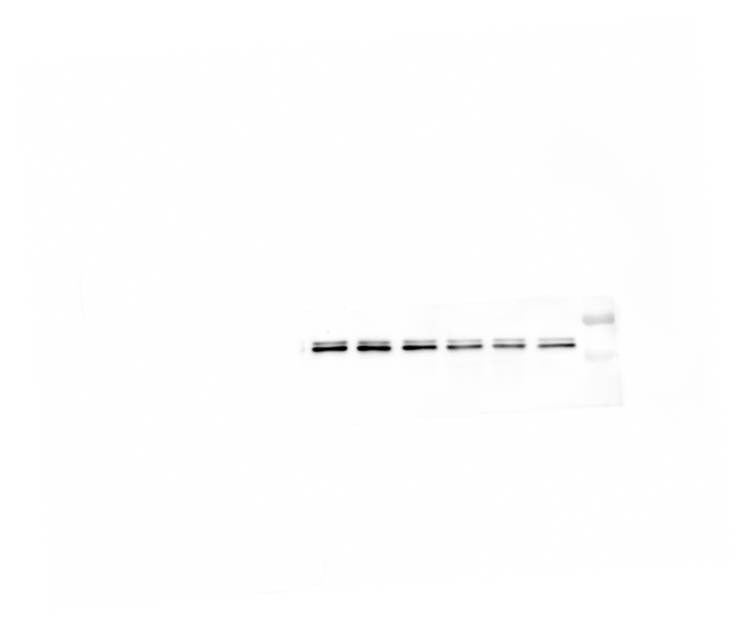

Supplement: Figure 6—source data 2. [file elife-98081-fig6-data2.zip › Figure6-source data 2/I/p-ERK.tif]

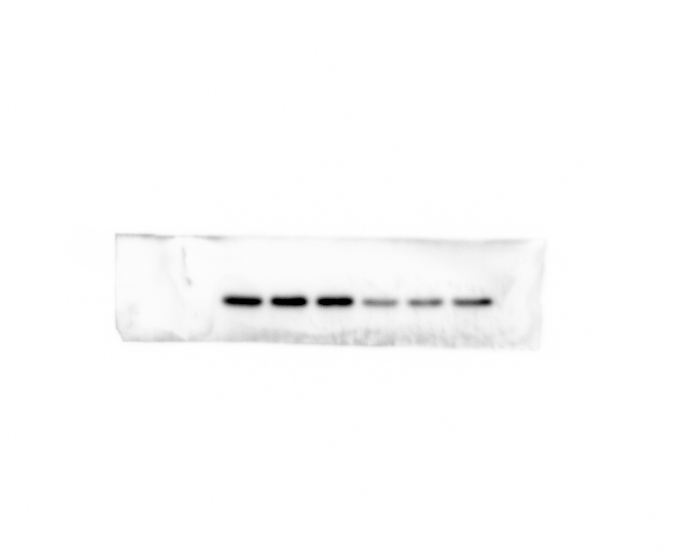

Supplement: Figure 6—source data 2. [file elife-98081-fig6-data2.zip › Figure6-source data 2/F/TTR.tif]

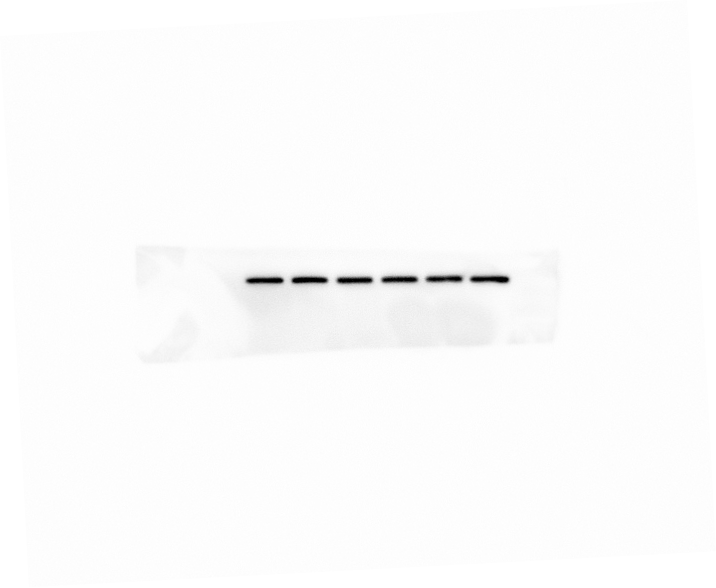

Supplement: Figure 6—source data 2. [file elife-98081-fig6-data2.zip › Figure6-source data 2/F/GAPDH.tif]

Figure 7A

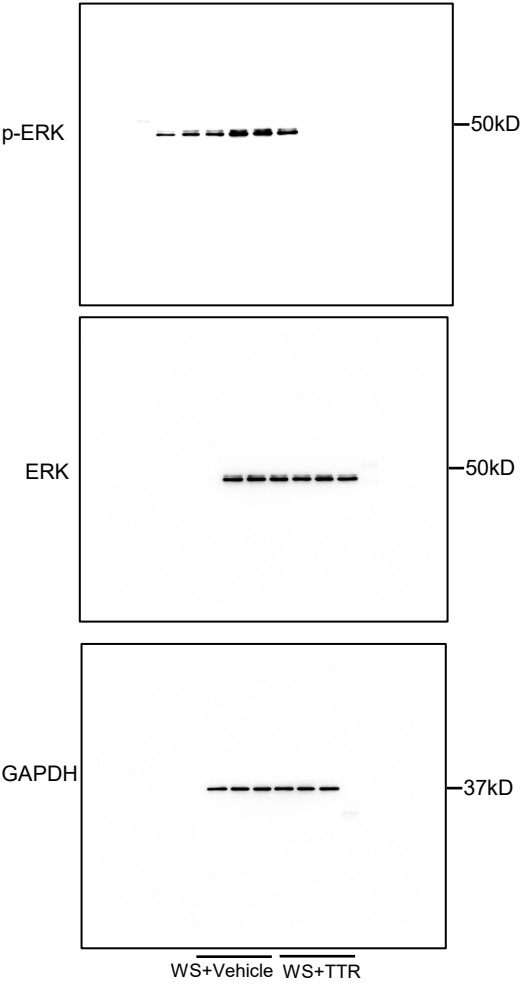

Figure 7D

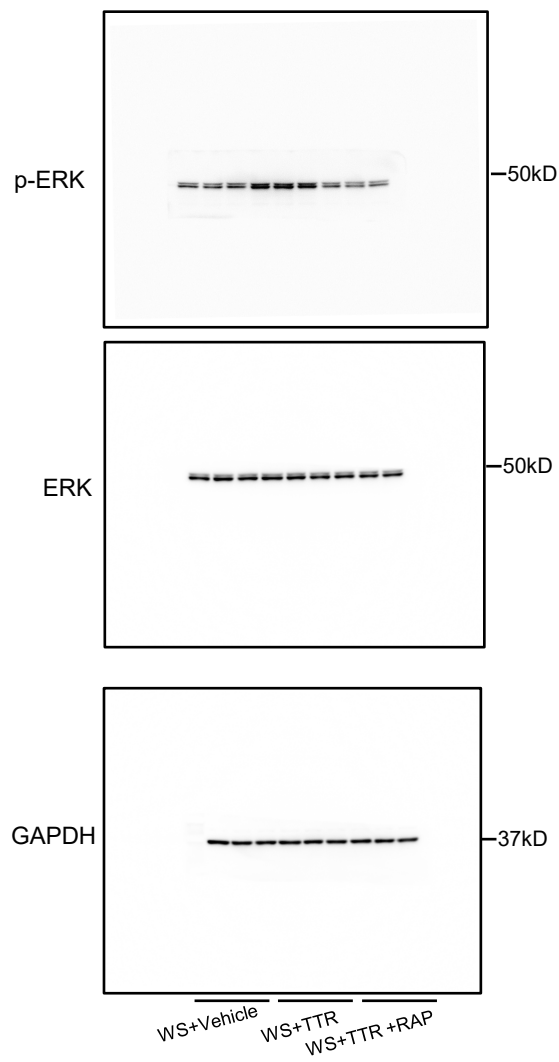

Supplement: Figure 7—source data 1. [file elife-98081-fig7-data1.zip › Figure 7-source data 1.pdf]

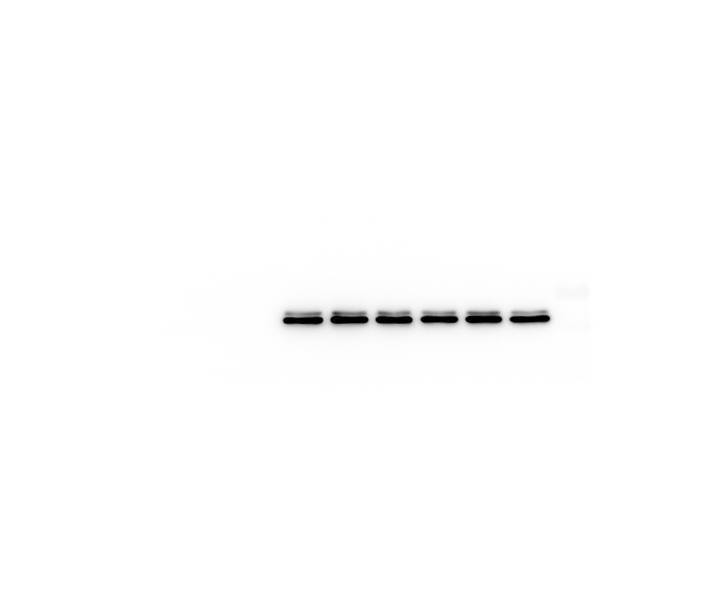

Supplement: Figure 7—source data 2. [file elife-98081-fig7-data2.zip › Figure7-source data 2/A/ERK.tif]

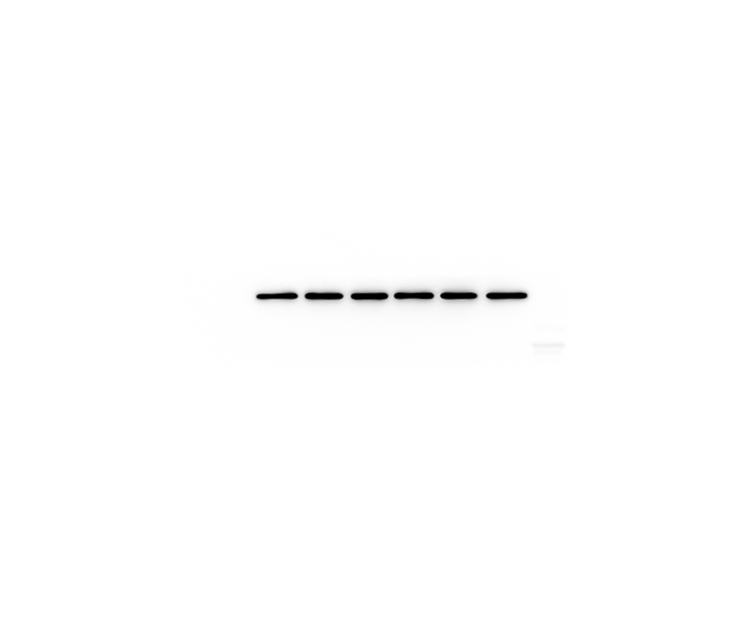

Supplement: Figure 7—source data 2. [file elife-98081-fig7-data2.zip › Figure7-source data 2/A/GAPDH.tif]

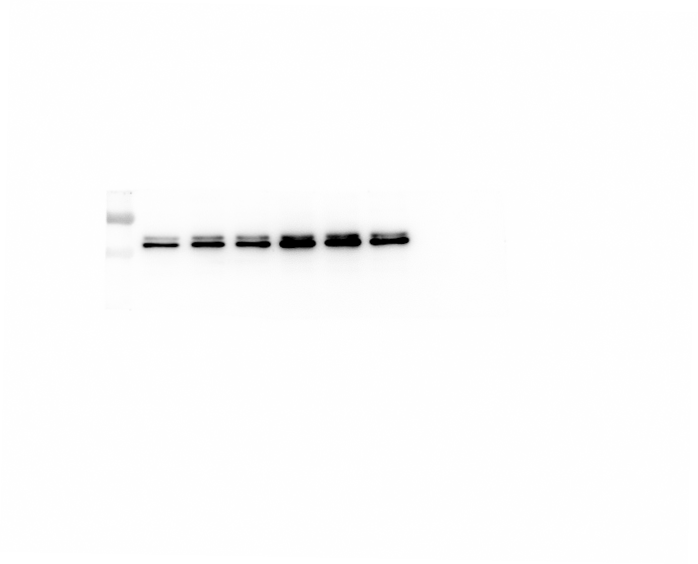

Supplement: Figure 7—source data 2. [file elife-98081-fig7-data2.zip › Figure7-source data 2/A/p-ERK.tif]

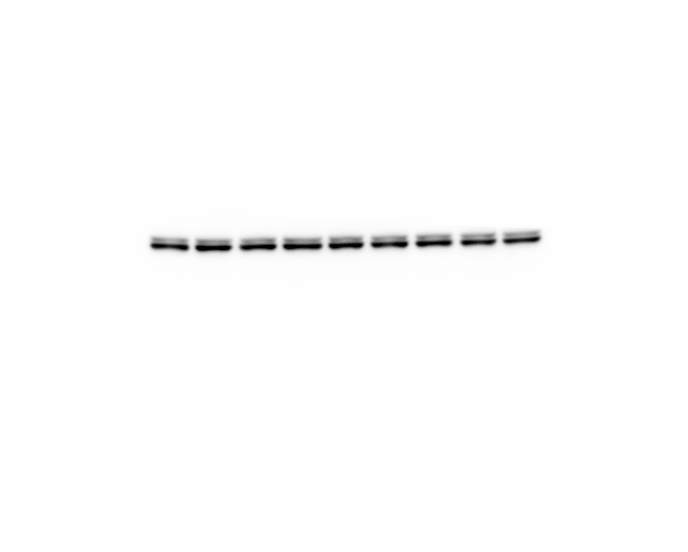

Supplement: Figure 7—source data 2. [file elife-98081-fig7-data2.zip › Figure7-source data 2/D/ERK.tif]

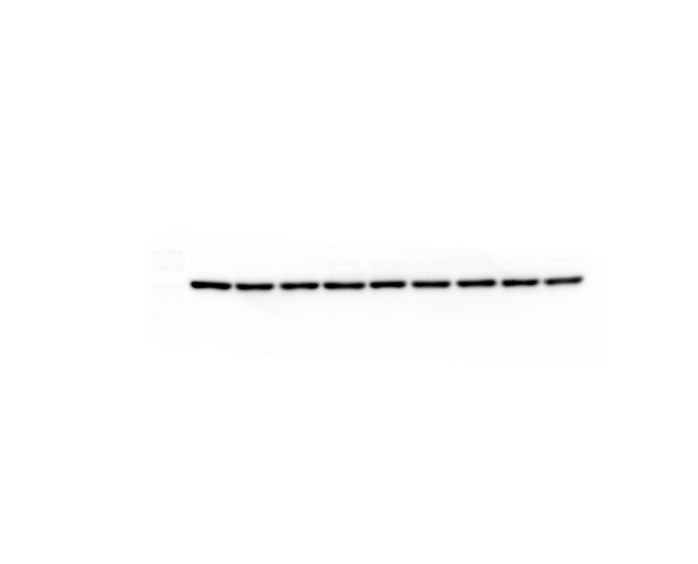

Supplement: Figure 7—source data 2. [file elife-98081-fig7-data2.zip › Figure7-source data 2/D/GAPDH.tif]

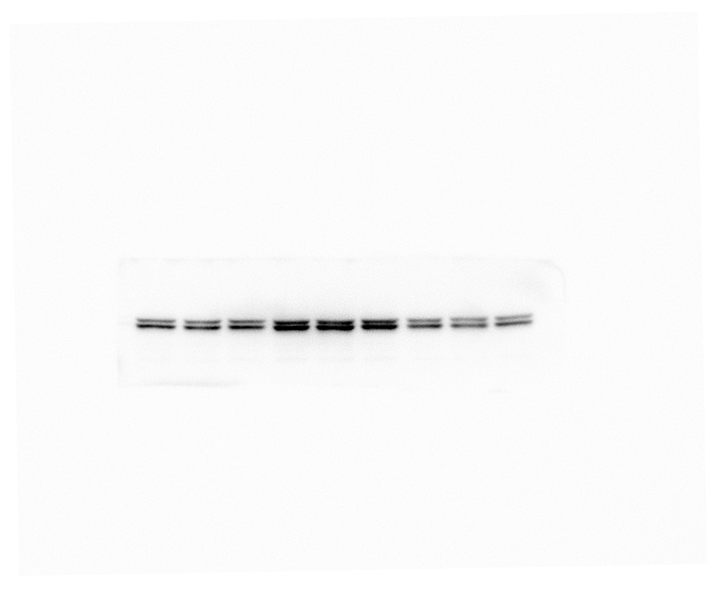

Supplement: Figure 7—source data 2. [file elife-98081-fig7-data2.zip › Figure7-source data 2/D/p-ERK.tif]

Figure 8J

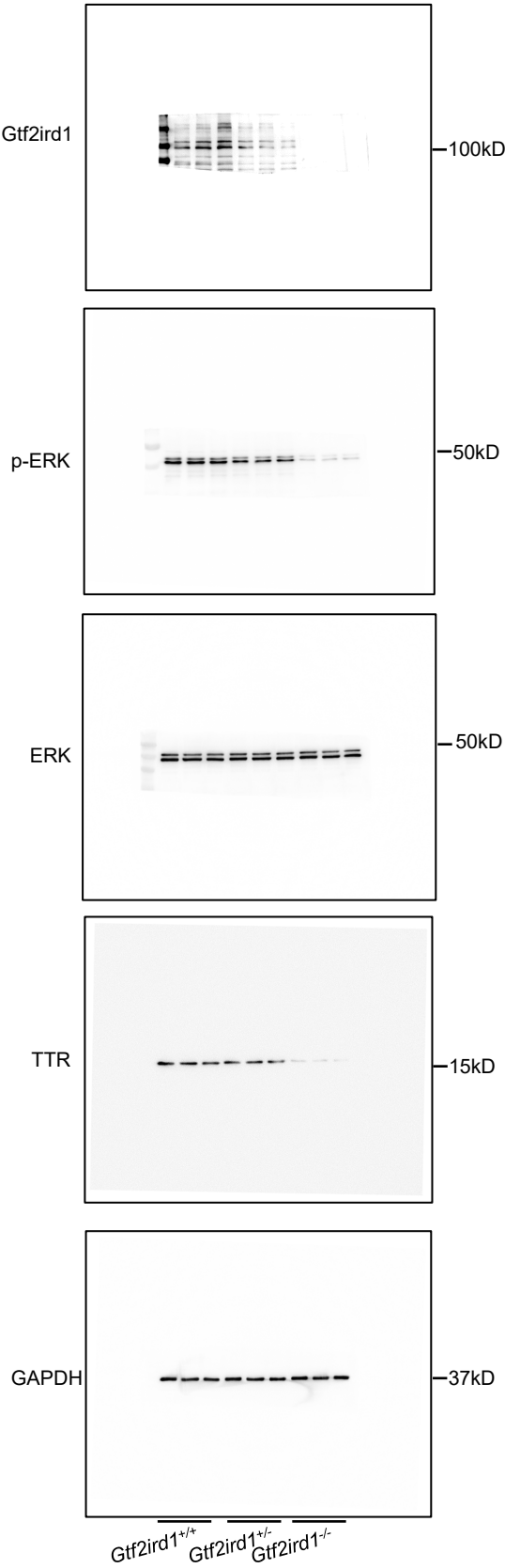

Supplement: Figure 8—source data 1. [file elife-98081-fig8-data1.zip › Figure 8-source data 1.pdf]

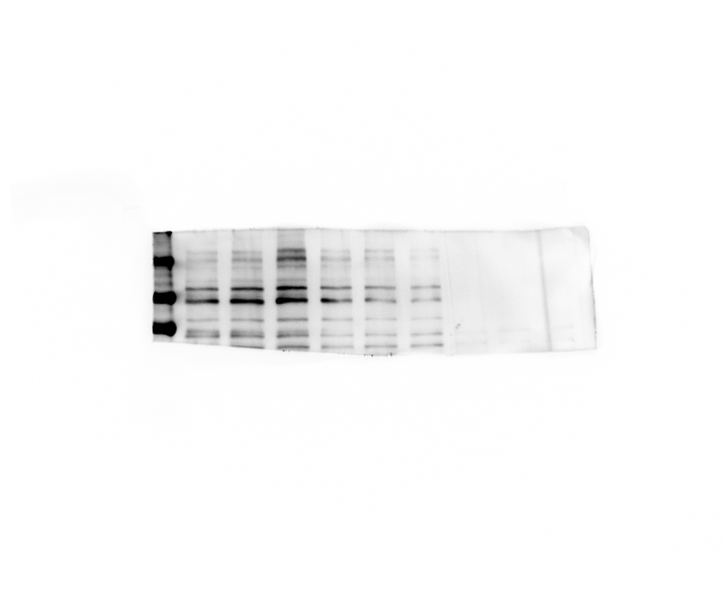

Supplement: Figure 8—source data 2. [file elife-98081-fig8-data2.zip › Figure8-source data 2/J/Gtf2ird1.tif]

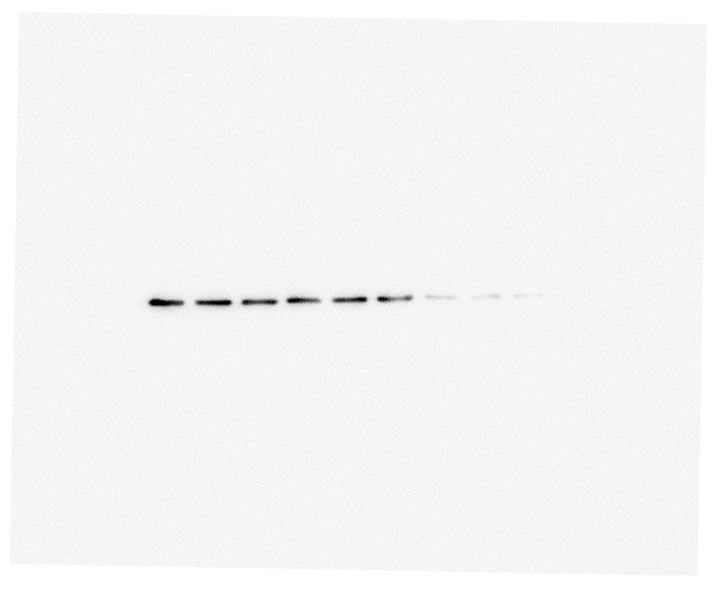

Supplement: Figure 8—source data 2. [file elife-98081-fig8-data2.zip › Figure8-source data 2/J/Ttr.tif]

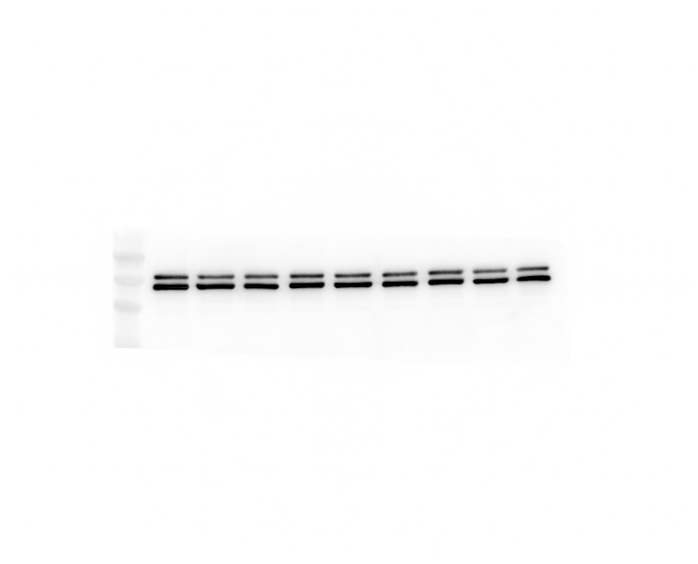

Supplement: Figure 8—source data 2. [file elife-98081-fig8-data2.zip › Figure8-source data 2/J/Erk.tif]

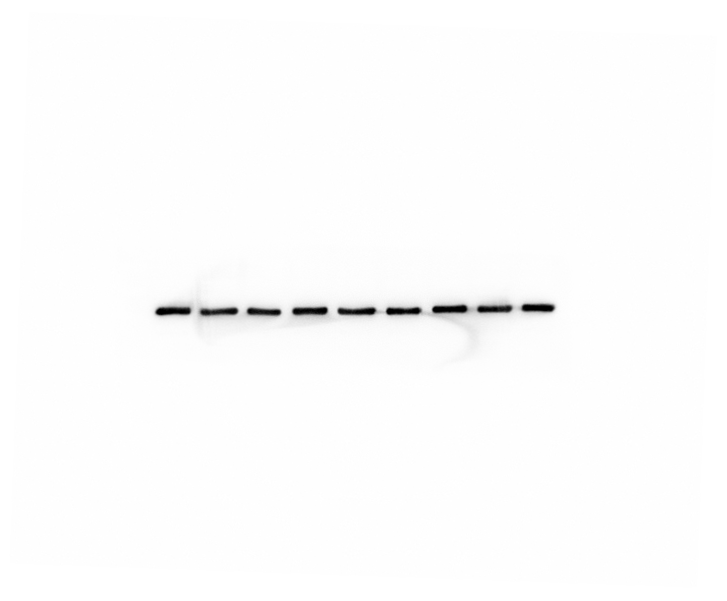

Supplement: Figure 8—source data 2. [file elife-98081-fig8-data2.zip › Figure8-source data 2/J/Gapdh.tif]

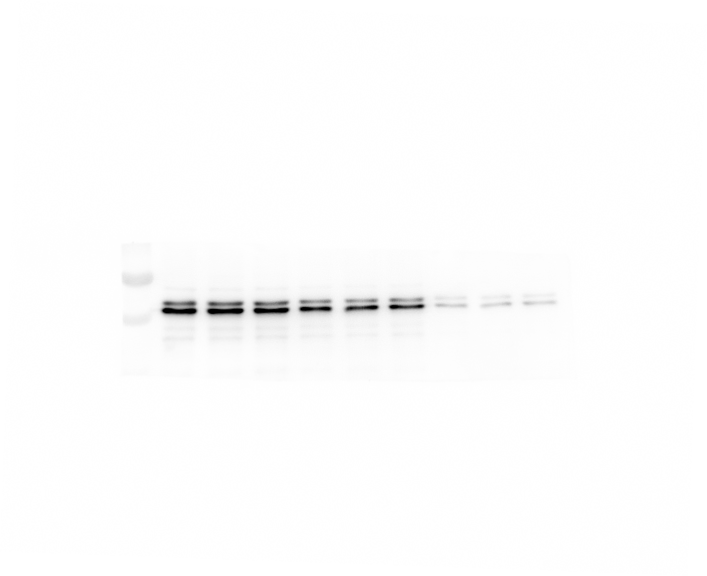

Supplement: Figure 8—source data 2. [file elife-98081-fig8-data2.zip › Figure8-source data 2/J/p-Erk.tif]

Figure 9J

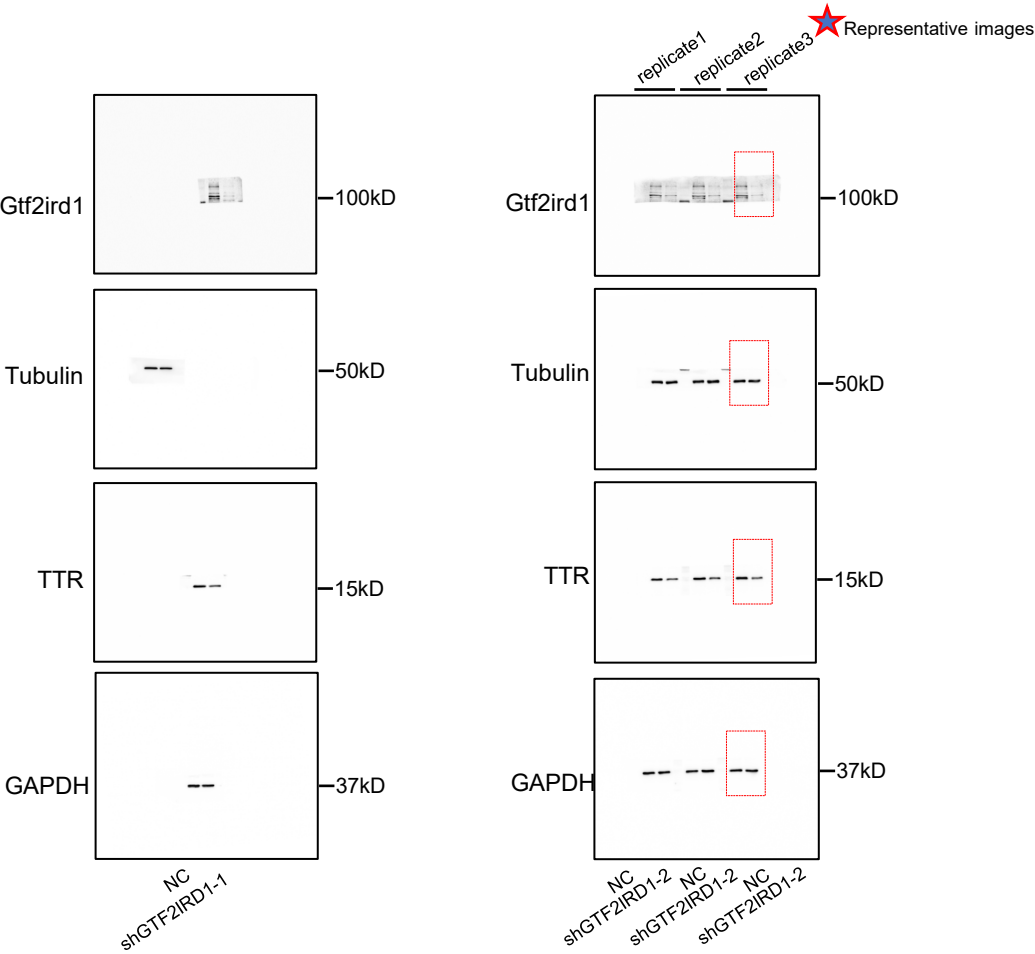

Supplement: Figure 9—source data 1. [file elife-98081-fig9-data1.zip › Figure 9-source data 1.pdf]

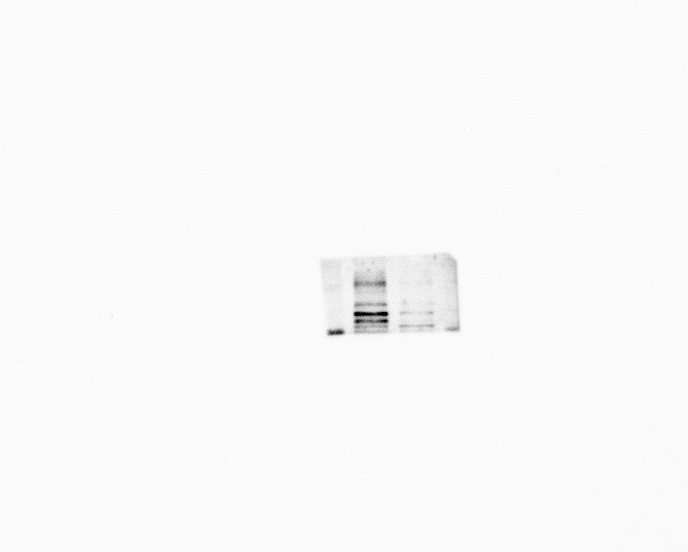

Supplement: Figure 9—source data 2. [file elife-98081-fig9-data2.zip › Figure9-source data 2/J/shGTF2IRD1-1/Gtf2ird1.tif]

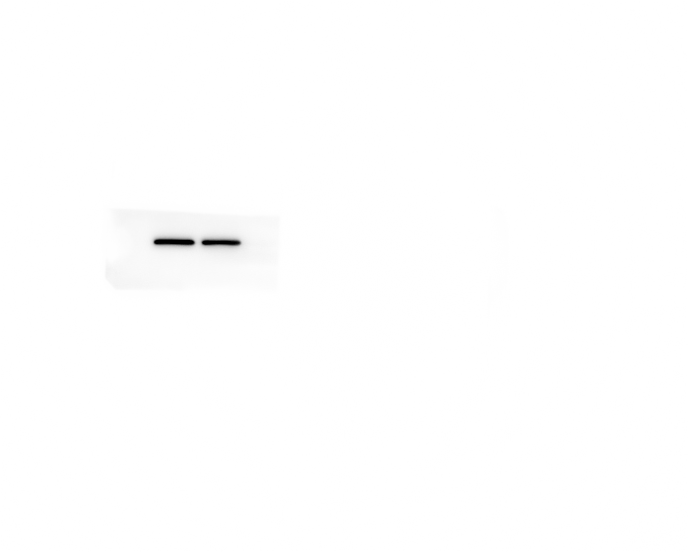

Supplement: Figure 9—source data 2. [file elife-98081-fig9-data2.zip › Figure9-source data 2/J/shGTF2IRD1-1/Tubulin.tif]

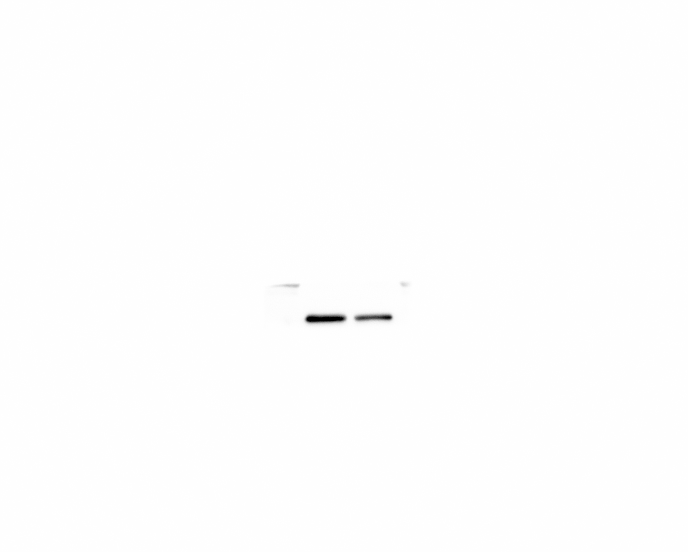

Supplement: Figure 9—source data 2. [file elife-98081-fig9-data2.zip › Figure9-source data 2/J/shGTF2IRD1-1/Ttr.tif]

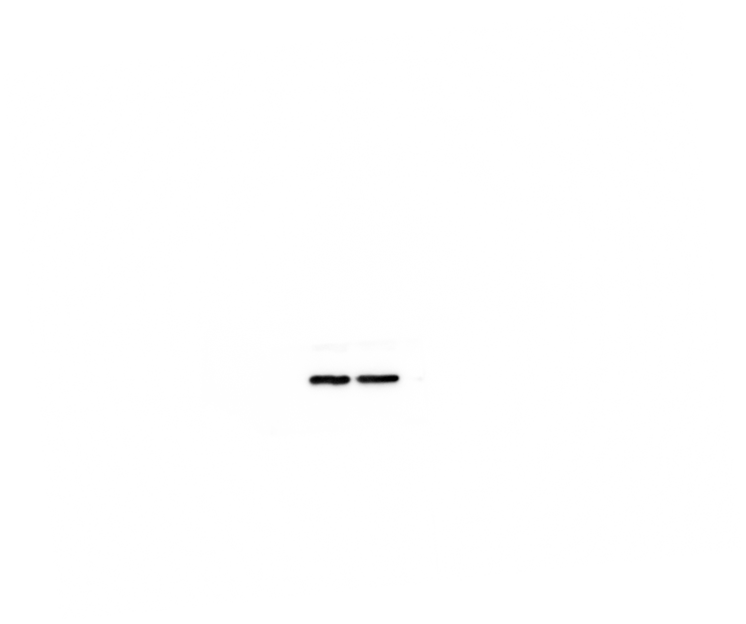

Supplement: Figure 9—source data 2. [file elife-98081-fig9-data2.zip › Figure9-source data 2/J/shGTF2IRD1-1/Gapdh.tif]

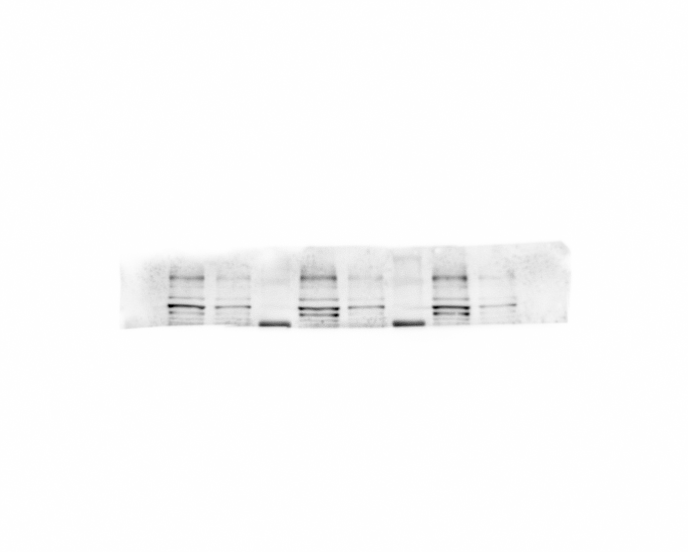

Supplement: Figure 9—source data 2. [file elife-98081-fig9-data2.zip › Figure9-source data 2/J/shGTF2IRD1-2/Gtf2ird1.tif]

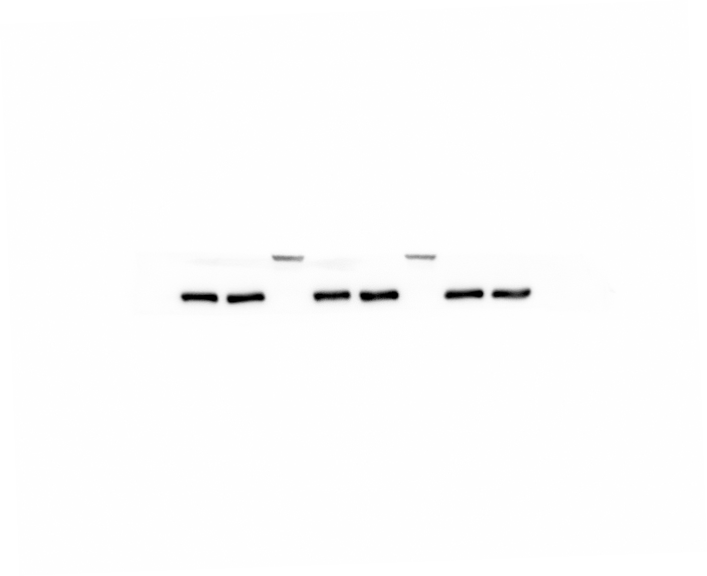

Supplement: Figure 9—source data 2. [file elife-98081-fig9-data2.zip › Figure9-source data 2/J/shGTF2IRD1-2/Tubulin.tif]

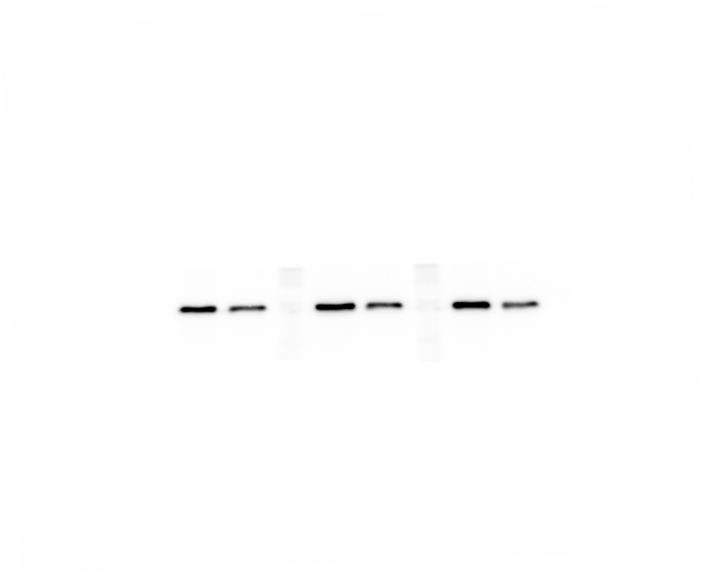

Supplement: Figure 9—source data 2. [file elife-98081-fig9-data2.zip › Figure9-source data 2/J/shGTF2IRD1-2/Ttr.tif]

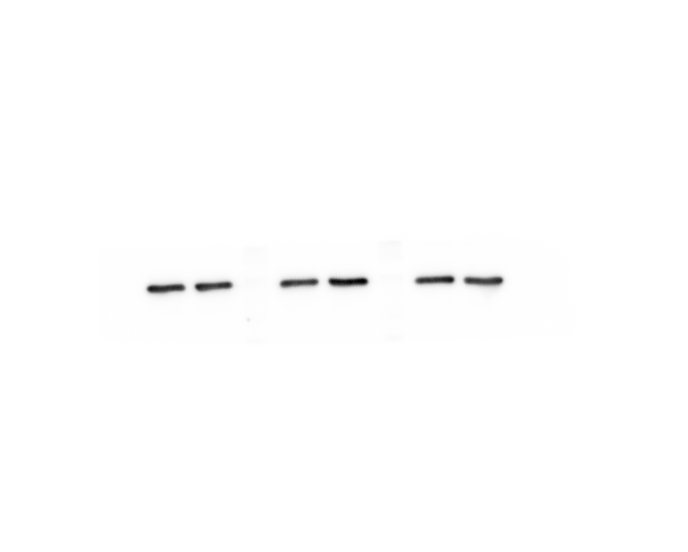

Supplement: Figure 9—source data 2. [file elife-98081-fig9-data2.zip › Figure9-source data 2/J/shGTF2IRD1-2/Gapdh.tif]
