# Supplementary material for: A human forebrain organoid model reveals the essential function of GTF2IRD1-TTR-ERK axis for the neurodevelopmental deficits of Williams syndrome
Source: eLife. 2024 Dec 13;13:RP98081. doi: 10.7554/eLife.98081 (PMC11643624; doi:10.7554/eLife.98081)
Supplement: Supplementary file 3. [file elife-98081-supp3.pdf]

CHIP-qPCR	
GAPDH-Fw	TCTGGTAACTCCGCCTTTGC
GAPDH-Rv	TACGGGATGGGTCTGAACGG
TTR-1-Fw	GACAGGTTCAAGCACGTCCA
TTR-1-Rv	GCTCCCCTTGAGGTGGATAAG
TTR-2-Fw	TGCACTAGCATTAGCGAGCA
TTR-2-Rv	CTGCGGTGCCATAGCTTACA
TTR-3-Fw	ATCCTGCCCGACCTTTGACT
TTR-3-Rv	ACATGTTTGAACAGGGGCCG
TTR-4-Fw	TGTGTTAGACACAGGGTTGGG
TTR-4-Rv	CTGCAGGCAGTGTCATACCC

RT-PCR MOUSE	
GTF2IRD1-Fw	TTCGTCCTCTAACCCAGAGTC
GTF2IRD1-Rv	ACAGAATTAGGGTGAAGTTCGGA
TTR-Fw	TCGTACTGGAAGACACTTGGC
TTR-Rv	CCAGAGTCGTTGGCTGTGAA
GAPDH-Fw	AGGTCGGTGTGAACGGATTTG
GAPDH-Rv	TGTAGACCATGTAGTTGAGGTCA
GTF2I-Fw	AAAGCCCTCCAATTTTCCTTTCA
GTF2I-Rv	TCCACACCTGATGCAGTAGTA
RT-PCR human	
OCT3/4-Fw	CCTCACTTCACTGCACTGTA
OCT3/4-Rv	CAGGTTTTCTTTCCCTAGCT
KLF4-Fw	GATGAACTGACCAGGCACTA
KLF4-Rv	GTGGGTCATATCCACTGTCT
CMYC-Fw	TGCCTCAAATTGGACTTTGG
CMYC-Rv	GATTGAAATTCTGTGTAACTGC
SOX2-Fw	CCCAGCAGACTTCACATGT
SOX2-Rv	CCTCCCATTTCCCTCGTTTT
HES5-Fw	AAGCACAGCAAAGCCTTCGTC
HES5-Rv	AGTAGCCTTCGCTGTAGTCCTG
TBR1-Fw	GAGGCAAATGGGTTCCTTGC
TBR1-Rv	TTTCTTGGCGCATCCAGTGA
PAX6-Fw	CCGAATTCTGCAGGTGTCCA
PAX6-Rv	GTCTGATGGAGCCAGTCTCG
TBR2-Fw	CTCACCAATAACAAAGGCGCA
TBR2-Rv	TGCAGTCGGGGTTGGTATTT
CTIP2-Fw	GATGACCACCTGCTCTCACC
CTIP2-Rv	GCTCATCTTTACCTGCAATGTTCT
TTR-Fw	CCGTGCATGTGTTCAGAAAGG
TTR-Rv	AGCTCTCCAGACTCACTGGT

GAPDH-Fw	GGAGCGAGATCCCTCCAAAAT
GAPDH-Rv	GGCTGTTGTCATACTTCTCATGG
18s-Fw	CGGCTACCACATCCAAAGGA
18s-Rv	GCTGGAATTACCGCGGCT
